# Synthesis of magnetic metal-organic framework (MOF) for efficient removal of organic dyes from water

**DOI:** 10.1038/srep11849

**Published:** 2015-07-07

**Authors:** Xiaoli Zhao, Shuangliu Liu, Zhi Tang, Hongyun Niu, Yaqi Cai, Wei Meng, Fengchang Wu, John P. Giesy

**Affiliations:** 1 State Key Laboratory of Environmental Criteria and Risk Assessment, Chinese Research Academy of Environmental Sciences, Beijing 100012, China; 2 State Key Laboratory of Environmental Chemistry and Ecotoxicology, Research Center for Eco-Environmental Sciences, Chinese Academy of Sciences, Beijing 100085, China; 3 Department of Veterinary Biomedical Sciences and Toxicology Centre, University of Saskatchewan, Saskatoon, Saskatchewan, Canada

## Abstract

A novel, simple and efficient strategy for fabricating a magnetic metal-organic framework (MOF) as sorbent to remove organic compounds from simulated water samples is presented and tested for removal of methylene blue (MB) as an example. The novel adsorbents combine advantages of MOFs and magnetic nanoparticles and possess large capacity, low cost, rapid removal and easy separation of the solid phase, which makes it an excellent sorbent for treatment of wastewaters. The resulting magnetic MOFs composites (also known as MFCs) have large surface areas (79.52 m^2^ g^−1^), excellent magnetic response (14.89 emu g^−1^), and large mesopore volume (0.09 cm^3^ g^−1^), as well as good chemical inertness and mechanical stability. Adsorption was not drastically affected by pH, suggesting π–π stacking interaction and/or hydrophobic interactions between MB and MFCs. Kinetic parameters followed pseudo-second-order kinetics and adsorption was described by the Freundlich isotherm. Adsorption capacity was 84 mg MB g^−1^ at an initial MB concentration of 30 mg L^−1^, which increased to 245 mg g^−1^ when the initial MB concentration was 300 mg L^−1^. This capacity was much greater than most other adsorbents reported in the literature. In addition, MFC adsorbents possess excellent reusability, being effective after at least five consecutive cycles.

The presence of dyes in effluents is a major concern due to their potential to cause adverse effects to flora, fauna and humans. Industries such as printing, paper, textile, electroplating, pulp mill, food and cosmetic, use dyes in order to color their products and generate considerable amounts of colored wastewater. Complex aromatic structures and xenobiotic properties of dyes make them more difficult to degrade^1^. Methylene blue (MB), a cationic dye, has been widely used in dying cotton, wood, and silk. It causes eye burns, which may be responsible for permanent injury to the eyes of wildlife and humans^2^. It can cause nausea, vomiting, profuse sweating, mental confusion, painful micturition, and methemoglobinemia if inhaled. Many methods have been developed in the decoloration of MB, such as adsorption, precipitation, reverse osmosis and ionexchange^3^,^4^,^5^. Adsorption is one of the most attractive approaches among these possible techniques due to its low cost, versatility and ease of operation. A variety of materials capable of removing MB have been reported^6^,^7^. Traditional absorbent materials have limitations in their application such as low adsorption capacity or difficulty being separated. There was thus a meed for an efficient and cost-effective adsorbent that presents large capacity, fast uptake rate and easy separation that could remove organic dyes, such as MB from wastewaters.

Recently, due to their new physicochemical properties compared with their single component analogues, hetero-structured nanocomposites have received attention from engineers^8^. Among various nanomaterials, metal-organic frameworks (MOFs), which are porous crystalline materials made of metal ions coordinated to organic ligands, have attracted significant attention due to their larger specific surface areas, easy separation, higher porosity, diversity of structures and functions^9^,^10^,^11^. MOFs built from various organic binding ligands and metal ions have allowed more systematic engineering of chemical and physical properties that make the useful for various applications in different fields including domains of gas adsorption and separation, sensors, drug delivery, catalysis or others^12^. MIL-101(Cr) exhibited greater sorption of benzene than activated carbon due to the larger pore diameter^13^. Hybrid zirconium-based MOF modified with cerium exhibited greater adsorption of NO_2_ under both moist and dry conditions^14^. When crystalline Cu_3_(BTC)_2_ was used as an electro-responsive, electro-rheological material dispersed in insulating oil it formed chain-like structures with excellent, controllable rheological properties.

Magnetic Fe_3_O_4_ can be easily separated from reaction liquids by use of an external magnetic field. Combinations of MOFs and magnetic nanoparticles of build magnetic MOF composites (also known as MFCs) have obvious advantages in adsorption and separation. Generally, preparation and application of MOFs requires centrifugation, which is laborious and inconvenient, their applications have been limited. Especially, controlling growth of MOF crystals on magnetic nanoparticles remains a challenge. There is limited literature that has reported syntheses of MFCs^15^,^16^,^17^. However, the procedure was time consuming because the reaction required two separate synthesis solutions for at least 25 repetitions each lasting 45 min^16^,^18^. Therefore, it was determined to be necessary to develop a more efficient method to prepare magnetic MOF composites.

Herein, we present a novel, simple and efficient scheme to fabricate magnetic MOF by simply controlling the rate of dropping of the organic ligand (Fig. 1). The magnetic MOF composite, Fe_3_O_4_/Cu_3_(BTC)_2_, was obtained by incorporation of Fe_3_O_4_ and Cu_3_(BTC)_2_. BTC is the acronym for benzene-1,3,5-tricarboxylate and the complex, Cu_3_(BTC)_2_, has been referred to in the literature as “HKUST-1”. MB was chosen as a typical adsorbate to be removed from water by use of and used Fe_3_O_4_/Cu_3_(BTC)_2_ as asorbent. Effects of pH, contact time, temperature and dosage on adsorption capacity were also investigated in detail. The Fe_3_O_4_/Cu_3_(BTC)_2_ exhibited both magnetic characteristics and high porosity, making it an excellent sorbent for treatment of wastewaters.

## Results and Discussion

**Figure 2:**
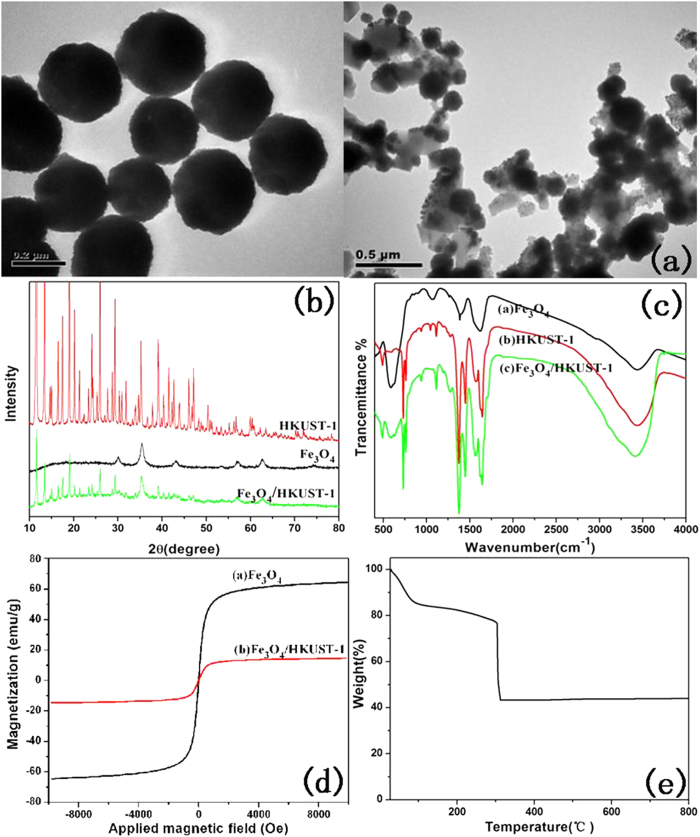
The structure and morphology of Fe_3_O_4_, Cu_3_(BTC)_2_ and Fe_3_O_4_/Cu_3_(BTC)_2_ nanocomposites. (**a**) TEM images. (**b**) XRD patterns. (**c**) FTIR spectra. (**d**) VSM curve. (**e**) Thermo gravimetric analysis (TGA) curve of Fe_3_O_4_/Cu_3_(BTC)_2_ under air atmosphere.

The structure and morphology of the Fe_3_O_4_/Cu_3_(BTC)_2_ was characterized and is described here. TEM images showed that Fe_3_O_4_ NPs were mono-dispersed, spherical with an approximate diameter of 200 nm and Fe_3_O_4_ (Fig. 2a) was encapsulated by a shell of Cu_3_(BTC)_2_ (Fig. 2b) and these two components of Fe_3_O_4_/Cu_3_(BTC)_2_ could be clearly identified. The network structure of Cu_3_(BTC)_2_ is an octahedron network constructed from dimer Cu paddle wheels linked by BTC^19^. The Cu^2+^ ions are connected through a weak bond and the residual axial coordination site is filled by a weakly bound water molecule. The BTC ligand combined these primary building blocks into a 3D octahedron network with an open pore system^20^.In this study, the functional MAA-Fe_3_O_4_ combined with free state Cu^2+^ ions first and the rate of nucleation by Cu_3_(BTC)_2_ could be controlled by the speed at which the organic ligand was added.

Peaks in XRD diffraction analysis of Fe_3_O_4_/Cu_3_(BTC)_2_ could be related to crystalline Fe_3_O_4_ (JCPDS file 19-0629) and Cu_3_(BTC)_2_^21^,^22^, respectively, and no peaks of impurities were detected, which indicates successful synthesis of Fe_3_O_4_/Cu_3_(BTC)_2_ (Fig. 2c). It was also confirmed that the composite is a real Fe_3_O_4_/Cu_3_(BTC)_2_ composite rather than a physical mixture of two separate phases of Fe_3_O_4_ and Cu_3_(BTC)_2_.

In FT-IR spectra of Fe_3_O_4_, peaks observed at 585 cm^−1^ are due to the Fe-O vibration (Fig. 2d)^23^. The adsorption peak at 1400 cm^−1^ is related to the vibration of COO- groups from citrate on the surface of Fe_3_O_4_, and the broad peak at 1626 cm^−1^ corresponds to the vibration of overlapping COO^−^ and H-O groups^24^. The Fe-O vibration was observed in both Fe_3_O_4_ and Fe_3_O_4_/Cu_3_(BTC)_2_, but the intensity of this peak decreased due to immobilization by the coating of Cu_3_(BTC)_2_. For Fe_3_O_4_/Cu_3_(BTC)_2_, the band at 1620 cm^−1^ and 1700 cm^−1^ suggests the presence of BTC^25^. The peaks observed at 1440 cm^−1^ and 930 cm^–1^were assigned to N-H vibration and in-plane bending vibration of O-H, respectively. The peak at 3434 cm^−1^ was due to surface-sorbed water and hydroxyl groups^22^. The band at 1570 cm^−1^was due to the C = C stretching vibrations of the aromatic ring of trimesic acid^26^. Intensities of peaks at 3375 and 3251 cm^−1^ are attributed to symmetric and asymmetric stretching of primary amines, respectively^19^.

All the materials exhibited super-magnetic characteristics (Fig. 2e). At 300 K, the magnetization saturation value of Fe_3_O_4_ was 59 emu g^−1^. Saturation magnetization of the microspheres decreased with the addition of Cu_3_(BTC)_2_ MOF, which can be explained by the increasing thickness of the nonmagnetic component. The Fe_3_O_4_/Cu_3_(BTC)_2_ exhibited super-paramagnetic properties at room temperature, indicating that, in the absence of an external magnetic field, they can distribute evenly in aqueous solution. The maximal saturation magnetization of Fe_3_O_4_/Cu_3_(BTC)_2_ was 14.89 emu g^−1^, which is sufficient for them to be isolated rapidly from large volumes of water samples by a strong Nd-Fe-B magnet.

The thermogram curve shows three different regions (Fig. 2f): (1) The first mass loss (14.5%) region between 30 and 90 °C indicating the loss of moisture; (2)The region between 90 and 304 °C with a slow loss of mass of 10% is related to loss of water from the MOF together with the oxidation of Cu^2+^ and FeO; (3) The third region starts at 304 °C, at which temperature the structure of Cu_3_(BTC)_2_ collapses and the organic linker is buried. This loss of mass is completed by 313 °C and then stable up to 800 °C. The stable residue was 43.3% of the original mass. The percent remaining after 310 °C could be regard as absolutely CuO and Fe_2_O_3_.

**Figure 3:**
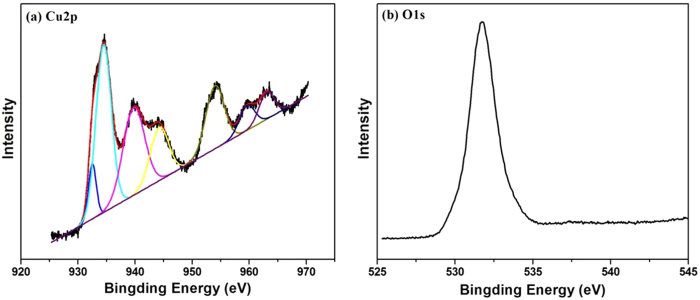
XPS spectra of (**a**) Cu2p region and (**b**) O1s region of the synthesized Fe_3_O_4_/Cu_3_(BTC)_2_.

XPS was employed to investigate the elemental composition of the surface of prepared composites (Fig. 3). In the 2*p* core level photoelectron spectra for Cu in Fe_3_O_4_/Cu_3_(BTC)_2_ (Fig. 3A), the peaks observed at 934.14 and 954.27 eV were related to Cu2p_3/2_ and Cu2p_1/2_ electrons, respectively. Similar satellite peaks were also observed in CuO, which indicates that Cu in the MOF is in the divalent form^27^, which is consistent with results of the FT-IR and XRD analyses. The binding energy of O1*s* core level was observed at 531.1 eV and corresponds to the characteristics of O^2+^ ions in the crystalline network (Fig. 3B). These results are in good agreement with results reported previously^28^.

**Figure 4:**
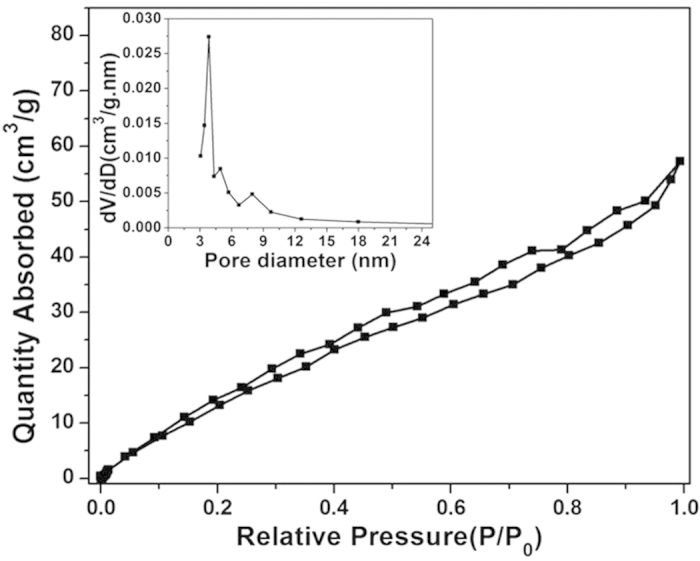
Nitrogen adsorption-desorption isotherm of Fe_3_O_4_/Cu_3_(BTC)_2_ (inset is the pore size distribution of Fe_3_O_4_/Cu_3_(BTC)_2_).

The porous properties and pore structure of particles of Fe_3_O_4_/Cu_3_(BTC)_2_ were investigated by measuring nitrogen adsorption isotherms (Fig. 4). The Brunauer–Emmett–Teller (BET) surface area and total pore volume of Fe_3_O_4_/Cu_3_(BTC)_2_ were determined to be 79.52 m^2^g^−1^ and 0.09 cm^3^g^−1^, respectively. The average pore size calculated from desorption in the N_2_ isotherm by Barrett-Joyner-Halenda (BJH) method was 4.4 nm. The properties of large specific surface area and high porosity could provide multiple accessible channels for MB immigrating.

### Mechanism of adsorption of MB on Fe_3_O_4_/Cu_3_(BTC)_2_

**Figure 5:**
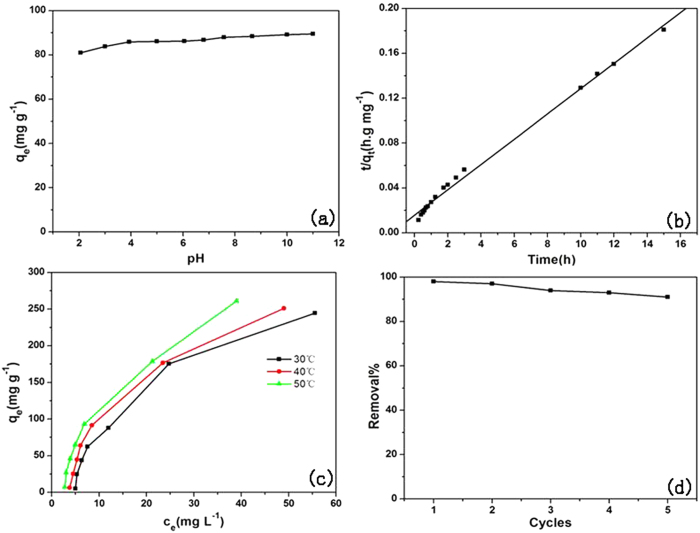
Mechanism of adsorption of MB on Fe_3_O_4_/Cu_3_(BTC)_2_ (**a**) Effect of pH on MB adsorption. (**b**) Effect of reaction time (the right one describes the pseudo-second-order kinetic) on MB removal. (**c**) Effect of solution temperature on MB removal. (**d**) Recyclability of Fe_3_O_4_/Cu_3_(BTC)_2_ for removing MB from aqueous solution.

Efficiencies of removal of MB were directly proportional to pH in the range of 2–11 (Fig. 5a). At greater pH, the surface of the adsorbent is negatively charged, which favors electrostatic interaction of cationic species of dye with the negatively charged surface. The electrostatic attraction force of MB with Fe_3_O_4_/Cu_3_(BTC)_2_ is likely to be greater at greater values of pH. Also, adsorption was not drastically affected by pH, suggesting the π-π stacking interaction and/or hydrophobic interactions between MB and Fe_3_O_4_/Cu_3_(BTC)_2_. These results are consistent with reports in the literature^7^,^29^,^30^.

Kinetics of adsorption of MB onto Fe_3_O_4_/Cu_3_(BTC)_2_ was analyzed using both the pseudo-first-order and pseudo-second order kinetic models. The pseudo-first-order kinetic model (Eq. 1)


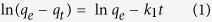


Where: *q*_*e*_ (mg g^−1^) and *q*_*t*_ (mg g^−1^) are the amounts of MB adsorbed at equilibrium and any time *t* (h), respectively, and *k*_*1*_ (h^−1^) is the adsorption rate constant.

The pseudo-second-order constants were calculated (Eq. 2).


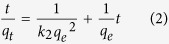


Where: *k*_*2*_ (g mg^−1^•h^−1^) is the pseudo-second-order rate constant.

Adsorption of MB was fast in the first 3 h, after which it increased slowly and reached a constant value after20 h (Fig. 5b). A similar trend was observed for adsorption of MB onto porous carbon nanospheres^31^ and tungstate oxide nanourchins^32^. The coefficient of determination (*R*) value of pseudo-second-order kinetic model was approximately 0.99 and the calculated *q*_*e,cal*_ (88 mg g^−1^) is very close to the experimental *q*_*e,exp*_ (84 mg g^−1^), demonstrating that the kinetics data fits well with the pseudo-second-order kinetic model. A similar phenomenon has been reported for adsorption of MB on wheat shells and activated carbon^33^,^34^.

The effect of temperature on adsorption of MB was studied by measuring adsorption at 303, 313 and 323K, respectively. Capacity for adsorption of MB to Fe_3_O_4_/Cu_3_(BTC)_2_ increased slightly with increasing temperature (Fig. 5c), which indicated that adsorption is controlled by an endothermic process.

Langmuir and Freundlich isotherm models were used to describe the relationship between adsorption of MB onto Fe_3_O_4_/Cu_3_(BTC)_2_ and its equilibrium concentration in water. The Langmuir model assumes that adsorption is monomolecular and occurs on a homogeneous surface with all the adsorption sites possessing identical affinities for the adsorbate, while the Freundlich isotherm model is often applicable to a heterogeneous adsorption surfaces with multilayer adsorption (Eq. 3 and 4 and Table 1).


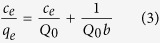


Where: *q*_*e*_ (mg g^−1^) is the amount of adsorbate adsorbed at equilibrium, *c*_*e*_ (mg L^−1^) is the equilibrium MB concentration, *Q*_*0*_ (mg g^−1^) and *b* (L mg^−1^) is the Langmuir constant related to the maximum adsorption capacity and the free energy of adsorption, respectively.


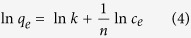


Where: *k* (mg g^−1^) is the Freundlich constant related to the adsorption capacity and *n* is the dimensionless exponent of the Freundlich equation.

Adsorption data were fitted well by the Freundlich isotherm model with a larger *R*^*2*^ value at all solution temperatures studied. All the *1/n* values were less than 1.0, demonstrating that adsorption of MB on the Fe_3_O_4_/Cu_3_(BTC)_2_ was favorable. The Freundlich model gives a better fit than the Langmuir model did, which is consistent with previously reported results^31^,^32^. Because the experimental data does not fit the Langmuir model well, the adsorption of MB by Fe_3_O_4_/Cu_3_(BTC)_2_ was compared to that of other adsorbents that have been reported in the literature. Capacities for adsorption, were calculated at certain initial concentrations instead of the monolayer adsorption value calculated by the Langmuir equation. When the initial concentration of MB was 30 or 300 mg L^−1^, capacity for adsorption of MB by Fe_3_O_4_/Cu_3_(BTC)_2_ was 25 and 244 mg g^−1^, respectively. These values were much greater than most of those obtained on other porous adsorbent materials that have been reported in the literature (Table 2). For activated carbon, the largest surface area is made up of micro pores with a radius of less than 1 nm, which are easily clogged in aqueous solution, which makes adsorptive sites unavailable to adsorbants. The mean size of pores on Fe_3_O_4_/Cu_3_(BTC)_2_ is 4.4 nm (meso pores). Therefore, MB can migrate from the solution through the pore channels to reach most of the potential adsorptive sites. This is one possible reason for the much greater capacity for adsorption of MB observed for Fe_3_O_4_/Cu_3_(BTC)_2_ than that of activated carbon. In addition, adsorption ability of Fe_3_O_4_/Cu_3_(BTC)_2_ was also compared with that of Cu_3_(BTC)_2_. WHEN the initial concentration of MB was 30 mg L^−1^, the adsorption capacity was 29.5 mg g^−1^, which was similar with Fe_3_O_4_/Cu_3_(BTC)_2_. Thus, it can be concluded that both total surface areas and pore size distributions affect the adsorption capacity of porous MOF-based materials. Effects of coexisting compounds on removal of MB were studied by using tap water instead of deionized water. When the initial concentration of MB was 30 or 300 mg L^−1^, capacity for adsorption of MB by Fe_3_O_4_/Cu_3_(BTC)_2_ was 20 and 241 mg g^−1^ in the tap water, respectively. This result indicated that coexisting compounds had little effect on absorption efficiency. Therefore, Fe_3_O_4_/Cu_3_(BTC)_2_ has great application potential in different water samples.

### Adsorption Thermodynamics Studies

Thermodynamic parameters including Gibbs free energy change (ΔG^0^), enthalpy (ΔH^0^), and entropy (ΔS^0^) were calculated for adsorption of MB onto Fe_3_O_4_/Cu_3_(BTC)_2_ at different temperatures (Eqs 5 and 6).


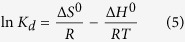



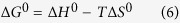


Where; *K*_*d*_ is the distribution coefficient, equal to *q*_*e*_/*c*_*e*_, *R* (8.314 JmoL^−1^ K^−1^) is the universal gas constant, *T* (K) is the temperature.

The negative value of Gibbs energy (△G^0^ was −3.68 kJ mol^−1^, −4.36 kJ mol^−1^ and −5.05 kJ mol^−1^ at 30 °C, 40 °C and 50 °C respectively) suggests that adsorption of MB on Fe_3_O_4_/Cu_3_(BTC)_2_ is spontaneous. The increase of absolute values of ΔG^0^ as a function of temperatures indicates that the adsorption is favorable at higher temperatures. The positive values of ΔH^0^ (17.01 kJ mol^−1^) and ΔS^0^ (68.27kJ mol^−1^) show that adsorption is endothermic and random at the solid-solution interface. If the value of ΔH^0^ is greater than 40 kJ moL^−1^, the process of adsorption is thought to proceed via chemisorption; while if the ΔH^0^ value is less than 40 kJ moL^−1^, adsorption is physic-sorption innature^35^. The value of ΔH^0^ observed in this study was 17 kJ moL^−1^, which indicates that MB adsorption on Fe_3_O_4_/Cu_3_(BTC)_2_ is likely due to physic-sorption.

### Reusability of adsorbent

To assess reusability of the absorbant, which contributes to reduce the cost of practical application process, methanol, ethanol and acetonitrile were used in desorption and regeneration experiment and acetonitrile shows the best desorption efficiency. Capacity for adsorption of MB on Fe_3_O_4_/Cu_3_(BTC)_2_ decreases slowly with increasing cycle number. A 90% removal rate of MB was achieved after five consecutive cycles, indicating the good recycling ability of Fe_3_O_4_/Cu_3_(BTC)_2_. (Fig. 5d).

## Conclusions

Nanoparticles of Fe_3_O_4_/Cu_3_(BTC)_2_ were prepared by a novel convenient method and used to remove MB from aqueous solution. This material exhibits excellent adsorption performance for MB attributing to the large, specific surface area and meso-porous channels. In addition, the Fe_3_O_4_/Cu_3_(BTC)_2_ adsorbent possessed excellent reusability, being effective after at least five consecutive cycles, indicating its potential for the purification of organic dyes water.

## Methods

### Synthesis of MAA-Fe_3_O_4_ nanoparticles

Particles of Fe_3_O_4_ were synthesized via a solvo-thermal method that has been reported previously^36^. First, 2.7 g FeCl_3_•6H_2_O, 1.0 g sodium citrate and 4.8 g NaAc were dissolved in 80 mL ethylene glycol with magnetic stirring for 0.5 h. Then the above mixed liquor was transferred to a sealed Teflon-lined stainless-steel which was heated in an autoclave at 200 °C for 10 h. The resultant Fe_3_O_4_ microspheres was collected, washed with ethanol several times, and dried at 50 °C under vacuum. Mercapto-acetic acid (MAA)-functionalized Fe_3_O_4_ nanoparticles were prepared as follows^18^, 0.5 g Fe_3_O_4_ was added to 100 mL of ethanol solution of mercapto-acetic acid (2.9 mM) under shaking for 24 h. The product was collected by an external magnetic field and washed.

### Synthesis of Fe_3_O_4_/Cu_3_(BTC)_2_

An aliquont of 0.2 g MAA-functionalized Fe_3_O_4_ microspheres were dispersed in 100 mL ethanol of 1.82 g Cu(NO_3_)_2_ and ultrasonicated for 30 min. Then, 100 mL of 0.875 g H_3_BTC was dropped into the above suspension at a constant rate of 1 mL min^−1^ followed by continuous mechanical stirring for 2 h. The resulting Fe_3_O_4_/Cu_3_(BTC)_2_ microspheres were collected from the reaction mixture under an external magnetic field, washed with ethanol several times, and dried at 50 °C under vacuum.

### Characterization

Several methods were used to characterize the particles. Morphology and particle size of the samples were determined by use of a transmission electron microscope (TEM; Hitachi H-7500, Japan) at 80 kV accelerated voltage. The crystal phase was investigated by use of a PAN analytical X’pert Pro diffractometer (XRD, Almelo, Netherlands) by using Cu Kα radiation ranging from 10° to 80° with a scan step of 0.02°. Thermo-gravimetric analysis (TGA) was carried out using a Q5000 TGA analyzer (TA Instruments, Japan) under a flow of air with a temperature ramp of 10 °C min^−1^. FTIR spectra were performed on a NEXUS 670 Infrared Fourier Transform Spectrometer (Nicolet Thermo, Waltham, MA). The specific surface area, pore size and volume were measured by BET methods (ASAP2000 V3.01A; Micromeritics, Norcross, GA). X-Ray photoelectron spectroscopy (XPS) was acquired with an ESCA-Lab-200i-XL spectrometer (Thermo Scientific, Waltham, MA) with monochromatic Al Kα radiation (1486.6 eV).The magnetic properties were analyzed on a LDJ9600 vibrating sample magnetometer (VSM, LDJ9600, Troy, MI).

### Batch Adsorption Experiments

Adsorption of MB on Fe_3_O_4_/Cu_3_(BTC)_2_ was performed in batch experiments. The optimal pH for adsorption of MB was determined by a series of experiments where the initial concentration of MB was maintained constant (100 mg L^−1^) at different pH values (pH 2–11). To evaluate the thermodynamic properties, adsorption isotherms were obtained by varying concentrations of MB from 10 to 300 mg L^−1^ at 303, 313 and 323 K, respectively. Solution pH was adjusted with 0.5 M NaOH or HCl. Kinetic studies were performed with an initial concentration of MB of 100 mg L^−1^. All batch tests were executed in 15 mL of polyethylene bottles by taking 10 mg, dm (dry mass) of Fe_3_O_4_/Cu_3_(BTC)_2_ with 10 mL of MB solution. Reactions proceeded in a temperature-controlled shaker at 200 rpm for 24 h. After equilibrium, the solid and solution were separated with a strong, Nd–Fe–B magnet and subjected to UV-Vis measurements. Parallel studies of adsorption were carried out in triplicate and the mean calculated for use in further calculations. Adsorption capacity was calculated (Eq. 7).


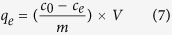


where *c*_*0*_ and *c*_*e*_ are the initial and equilibrium concentrations of MB (mgL^−1^), respectively, *m*is the mass of dry adsorbent (g)and *V* is volume of the solution (L).

Regeneration of the exhausted Fe_3_O_4_/Cu_3_(BTC)_2_ saturated with MB was examined by using solvent desorption techniques. Methanol, ethanol and acetonitrile were as the eluent to regenerate Fe_3_O_4_/Cu_3_(BTC)_2_. The eluent solution was added to the used Fe_3_O_4_/Cu_3_(BTC)_2_ and the mixture was ultrasonicated for 30 min.

Concentrations of MB in the supernatant solution were measured before and after adsorption was determined using a UV-vis spectrophotometer at 660 nm. The supernatant from the Fe_3_O_4_/Cu_3_(BTC)_2_ did not exhibit any absorbance at this wavelength and the calibration curve was reproducible and linear over the concentration range used in this work.

## Additional Information

**How to cite this article**: Zhao, X. *et al.* Synthesis of magnetic metal-organic framework (MOF) for efficient removal of organic dyes from water. *Sci. Rep.* **5**, 11849; doi: 10.1038/srep11849 (2015).

## Figures and Tables

**Figure 1:**
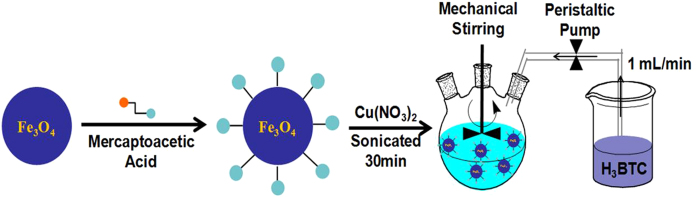
Synthesis of Fe_3_O_4_/Cu_3_(BTC)_2_ magnetic materials.

**Table 1:** Langmuir and Freundlich isotherms parameters for MB adsorption on Fe_3_O_4_/Cu_3_(BTC)_2_.

Parameters	Solution temperature (K)		
Isotherm	303	313	323
Langmuir:			
*Q*_*0*_/mg g^–1^	769.2308	666.6667	555.5556
*b*/L mg^–1^	0.009207	0.013562	0.022756
*R*^*2*^	0.2928	0.3981	0.7318
Freundlich:			
*K*/mg g^–1^ (L mg^–1^)^1/n^	7.961361	11.00335	15.18791
*1/n*	0.9053	0.8485	0.8076
*R*^*2*^	0.9171	0.8952	0.9432

**Table 2:** Comparison of capacities of adsorption of MB on different adsorbents.

Materials	*Q*_*e*_(mg g^−1^)	*Q*_*e*_(mg g^−1^)	Ref
	*C*_*0*_ = 30 mg L^−1^	*C*_*0*_ = 300 mg L^−1^	
Fe_3_O_4_/ Cu_3_(BTC)_2_	25	244	This work
Cu_3_(BTC)_2_	29.5	248	This work
Chitosan–clay composite	20	200	3
Acid modified local clay beads	15	150	4
Carbon nanotubes	24	–	2
Coir pith carbon	6	–	37
Graphene oxide	31	–	5
Bamboo based activated carbon	–	260	38
Rattan sawdust based activated carbon	–	220	33
Groundnut shell activated carbon	–	167	6
MOF-235	100	180	39
Hierarchically mesostructured MIL-101	21	–	40
Amino-MIL-101(Al)	380	762	41
